# Fine-Tuning Electrokinetic Injections Considering Nonlinear Electrokinetic Effects in Insulator-Based Devices

**DOI:** 10.3390/mi12060628

**Published:** 2021-05-28

**Authors:** Abbi Miller, Nicole Hill, Kel Hakim, Blanca H. Lapizco-Encinas

**Affiliations:** Microscale Bioseparations Laboratory and Biomedical Engineering Department, Rochester Institute of Technology, Rochester, NY 14623, USA; alm7175@g.rit.edu (A.M.); nsh3709@rit.edu (N.H.); ksh9238@g.rit.edu (K.H.)

**Keywords:** electrokinetics, electrophoresis, nonlinear electrokinetics, electrokinetic injection, microfluidics

## Abstract

The manner of sample injection is critical in microscale electrokinetic (EK) separations, as the resolution of a separation greatly depends on sample quality and how the sample is introduced into the system. There is a significant wealth of knowledge on the development of EK injection methodologies that range from simple and straightforward approaches to sophisticated schemes. The present study focused on the development of optimized EK sample injection schemes for direct current insulator-based EK (DC-iEK) systems. These are microchannels that contain arrays of insulating structures; the presence of these structures creates a nonuniform electric field distribution when a potential is applied, resulting in enhanced nonlinear EK effects. Recently, it was reported that the nonlinear EK effect of electrophoresis of the second kind plays a major role in particle migration in DC-iEK systems. This study presents a methodology for designing EK sample injection schemes that consider the nonlinear EK effects exerted on the particles being injected. Mathematical modeling with COMSOL Multiphysics was employed to identify proper voltages to be used during the EK injection process. Then, a T-microchannel with insulating posts was employed to experimentally perform EK injection and separate a sample containing two types of similar polystyrene particles. The quality of the EK injections was assessed by comparing the resolution (*Rs*) and number of plates (*N*) of the experimental particle separations. The findings of this study establish the importance of considering nonlinear EK effects when planning for successful EK injection schemes.

## 1. Introduction

Microfluidic devices have made significant impacts on several fields, including bioanalysis and clinical assessments. Working on the microscale offers attractive characteristics, such as a fast response time, portability, and a high level of integration. Electrokinetics (EK) is one of the main branches of microfluidics due to its flexibility and ease of application, since a single applied voltage can drive both the liquid and bioparticles present in a sample [1,2]. In recent years, there has been significant progress in the development of microscale electrokinetic EK methodologies for the identification, separation, enrichment, analysis, and detection of a wide array of bioparticles, ranging from macromolecules to parasites [3,4,5,6]. Furthermore, EK devices offer the unique potential of being able to exploit both linear and nonlinear EK phenomena [7,8] within the same system, leading to highly selective and discriminatory separation and purification processes [9,10].

An essential element of any EK-based system is sample injection [11], as the quality and resolution of any EK separation process greatly depends on the conditions of the injection system. Reproducibility, efficiency, and sample focusing are crucial aspects in EK injection systems. Important reports have been dedicated to reviewing the advances in EK sample injection [11,12] in comparison to hydrodynamic sample injection [13]. As a result, unique and novel EK injections schemes have been developed, and many of them have been applied to microchannel electrophoretic systems, also called microchip electrophoresis. Generally, EK injection systems are able to deliver a fixed volume of sample to the main channel. A plethora of new configurations featuring a wide array of channels with T-shaped intersections have been developed [12,14]. Two commonly used EK injection methods are pinched injection, which involves two steps (injection and separation), and gated injection, which involves three steps (loading, gating, and injection) [14,15,16]. The second EK injection method was the one used in this study. The gated injection process, employing a single T-cross four-reservoir channel, starts by introducing the particle sample into the sample reservoir while the other three reservoirs are filled with the buffer or suspending media. The loading step is when the particle sample solution migrates from the sample reservoir to the sample waste reservoir, filling the entire length of the channel that is perpendicular to the separation channel with sample. A key component of the loading step is to fill out the intersection of the T-device with particle solution. Next, in the gating step, a plug of the particle sample is first introduced into the separation channel—that is, the applied voltages cut off a defined volume of the particle sample and force this defined sample plug to migrate into the separation channel and post array. Lastly, the injection step is mainly responsible for pushing the plug of the particle sample across the length of the separation channel towards the outlet reservoir. The voltages applied during the injection step are the voltages associated with the particle separation process.

Pinched injections can provide accurate, well-defined sample volumes that can enable highly efficient separations; however, microdevice dimensions limit the injection volume. This drawback can be overcome with gated injections [14,15,16]. Gated injection schemes were first proposed by Jacobson et al. [17], and they allow for the control of the sample volume by varying the injection time and velocity. Since then, numerous schemes have been developed for a large number of specific applications that range from injecting small molecules [18] to injecting cells [19]. Batalla et al. [18] used an optimized protocol in a glass microchip that featured simultaneous injections from two distinct reservoirs followed by electro-focusing prior to separation in the main channel. They employed this system for the detection of D-amino acid enantiomers that act as biomarkers. Zhang et al. [19] employed a glass microdevice to carry out single-cell gated injections in a three step process. This group was able to selectively inject a single human liver cancer (HepG2) target cell from a sample (at a concentration of 10^5^ cells/mL) into the channel for the analysis of hydrogen peroxide content. The authors pointed out that cell concentration in the sample was important, as low concentrations (~10^4^ cells/mL) made the injection difficult and too high concentrations (10^6^ cells/mL) clogged the microchannel.

The above-mentioned reports illustrate the growing interest in the development of improved EK injection schemes that can be tailored for specific applications and offer a high level of flexibility. To develop superior EK injection methods, a full understanding of the EK phenomena and associated governing principles is required. For example, when comparing EK injection with hydrodynamic injection [20], it is important to note the hydrodynamic injection is not biased, while EK injection is biased on analyte electrophoretic mobility [16]. The great majority of EK injection schemes have been designed for capillary systems or microchannel systems that do not feature any type of structure within the capillary or the microchannel. The present study was focused on the development of optimized EK injection schemes for direct current insulator-based EK (DC-iEK) systems [7,8]. These are microchannels that contain arrays of insulating structures, and the presence of these structures generates a nonuniform distribution of the electric field when a potential is applied across the channel [21,22]. Nonlinear EK effects are enhanced in DC-iEK systems since the area between posts become regions of higher electric field intensity; therefore, when injecting samples into an iEK channel, nonlinear effects must be considered. These effects are essential for determining the appropriate voltage schemes in an EK injection, as these voltages affect the overall quality of the final particle separation; that is, a poorly executed injection will produce a poor separation. This is especially critical for the second step of the injection, gating, as it is when the particles are first introduced to the post array (separation channel) where the particle experience greater electric fields induced by the constrictions of the post array. Recently, there was a major discovery in the field of DC-iEK systems, as it was demonstrated that dielectrophoresis (DEP) was not the major EK force responsible of particle trapping and manipulation in these systems [7,23]. Particle trapping and enrichment in DC-iEK are mainly the result of the balance between electroosmosis (EO) and linear and nonlinear electrophoresis (EP^(1)^ and EP^(3)^, respectively) [7,23]. There are two main particle EK migration regimes in iEK systems: particle streaming and particle trapping. Particle streaming is particle migration primarily under linear EK effects, and particle trapping is when particle migration is stalled by nonlinear EK effects and bands of trapped particles are formed leading to significant enrichment [24]. The potential of iEK systems is significant, as their applicability for separating and analyzing valuable particles, including protein particles [25], nanovesicles [26], viruses [27], cells [8,28,29], and micro and nanoparticles [30,31], has been fully demonstrated. Given this new knowledge, the present study was focused on designing EK sample injection schemes for DC-iEK systems while considering the effects of nonlinear EP (known as the EP of the second kind or EP^(3)^). An elongated T-cross iEK channel with asymmetrical insulating posts made from polydimethylsiloxane (PDMS) was employed to perform EK injections and separate a sample containing two types of polystyrene particles, each with a distinct size and electrical charge. A longer channel with an extended post array was chosen because previous work has shown that lengthening the post array can allow for successful particle separation under a particle streaming regime. Essentially, elongated insulating post arrays allow for the exploitation of small differences in particle electrokinetic mobilities, thus enabling particles to gradually separate as they migrate through the separation channel (as shown by the chromatographic technique used by Hill and Lapizco-Encinas) [24]. By employing simulations with COMSOL Multiphysics and experimentation, it was demonstrated that it is essential to consider the effects of EP^(3)^ on particle migration when designing an EK injection process for a DC-iEK device. In particular, optimizing the gating step of an EK gated injection process proved to be essential for ensuring a successful EK injection. These findings establish the importance of considering nonlinear EK effects when planning for successful EK injection schemes.

## 2. Theory

**Figure 1:**
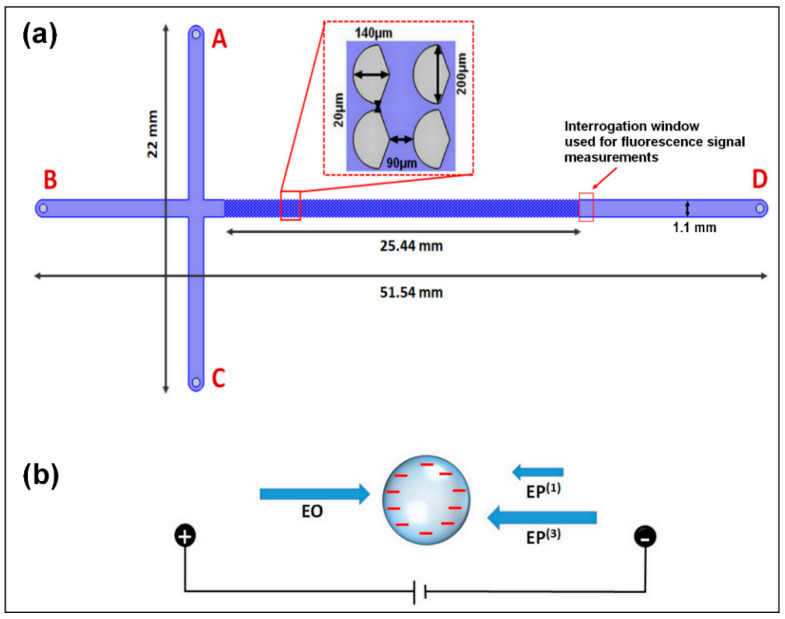
(**a**) Schematic representation of the microchannel employed, illustrating the dimensions of the channel and the asymmetric insulating posts. The device has a total of four reservoirs labelled A–D, where A is the reservoir where the sample was introduced. (**b**) Representation of the three main EK phenomena acting on the negatively charged microparticles in this system.

For the present study, we employed an insulator-based “T” device, which is depicted in Figure 1a. There were several EK phenomena acting on the particles in our devices, including EO, EP^(1)^, EP^(3)^, and DEP. At lower electric fields, linear EK, the superposition of EO and EP^(1)^ dominates particle migration. The expressions for the linear EK phenomena are as follows [7]:

(1) $$v_{EO}=\mu_{EO}E,$$

(2) $$v_{EP}^{\left(1\right)}=\mu_{EP}^{\left(1\right)}E,$$

(3) $$v_{EK}=\mu_{EK}E=\left(\mu_{EO}+\mu_{EP}^{\left(1\right)}\right)E,$$

where $v_{EO}$, $v_{EP}^{\left(1\right)}$, and $v_{EK}$ are the EO, linear EP^(1)^, and linear EK velocities, respectively; E is the electric field; and $\mu_{EO}$, $\mu_{EP}^{\left(1\right)}$, and $\mu_{EK}$ are the respective mobilities. These mobilities depend on the particle zeta potential ($\zeta_{P}$) and the zeta potential of the channel wall ($\zeta_{W}$), as well as on media viscosity (*η*), and media permittivity ($\varepsilon_{m}$):

(4) $$\mu_{EO}=-\frac{\varepsilon_{m}\zeta_{W}}{\eta },$$

(5) $$\mu_{EP}^{\left(1\right)}=\frac{\varepsilon_{m}\zeta_{P}}{\eta },$$

At higher magnitudes of the electric field, nonlinear EK phenomena become dominant. In the case of the systems employed in this study, the considered nonlinear phenomena were EP^(3)^ and DEP. The expressions for the EP^(3)^ and DEP velocities are [7,32,33]:

(6) $$v_{EP}^{\left(3\right)}=\mu_{EP}^{\left(3\right)}\left(E\cdot E\right)E,$$

(7) $$v_{DEP}=\mu_{DEP}\nabla E^{2},$$

where $v_{EP}^{\left(3\right)}$ and $v_{DEP}$ are the EP^(3)^ and DEP velocities, respectively; $\mu_{EP}^{\left(3\right)}$ and $\mu_{DEP}$ are the mobilities; and $\nabla E^{2}$ represents the electric field gradient. Considering all four EK phenomena, the overall particle velocity in iEK systems becomes:

(8) $$v_{P}=v_{EO}+v_{EP}^{\left(1\right)}+v_{EP}^{\left(3\right)}+v_{DEP},$$

The present study was focused on developing a strategy for successful EK injections while considering the effects of EP^(3)^ for systems with insulating structures within the separation channel. Particle migration was modeled by employing Equation (8), and we assessed the effect of EP^(3)^ on the EK injection and, thus, the quality of the separation of a mixture containing two types of distinct particles. The quality of the particle separations was evaluated in terms of the separation resolution (*Rs*) and number of plates (*N*) by employing the following expressions, where *t_R_* is retention time and *W* is the width at the base of the peak in an electropherogram.

(9) $$Rs=\frac{2\left(t_{R2}-t_{R1}\right)}{W_{1}+W_{2}},$$

(10) $$N=\frac{16\;{t_{R}}^{2}}{W^{2}}.$$

## 3. Materials and Methods

### 3.1. Microdevice Fabrication

Standard soft lithography techniques were employed to create the PDMS microchannel design shown in Figure 1a. The details of the process can be found in another study by our group [21]. All internal walls of the microchannel, including the insulating post surfaces, were made from PDMS, thus ensuring the same wall zeta potential ($\zeta_{W}$) across the device and resulting in a consistent EO flow. A device with asymmetric insulating posts was selected for this study, since previous reports have demonstrated that asymmetric posts, in particular those with diamond shapes, offer high discriminatory capabilities when differentiating particles by their electromigration velocities [24,34]. The four reservoirs in the channels were created using a 4-mm diameter biopsy punch before the cured PDMS device was plasma bonded to the PDMS-coated glass wafer.

### 3.2. Suspending Media and Microparticle Samples

The suspending media comprised a buffer solution of 0.2 mM K_2_HPO_4_ with a conductivity of 41 µS/cm and a pH of 7.33. This suspending media also contained 0.05% (*v/v*) of Tween 20, which was added to prevent particle clumping and sticking. The wall zeta potential ($\zeta_{W}$) in our devices was measured by employing a refined current monitoring methodology developed in our laboratory [35]. This wall zeta potential value was determined to be −60.1 mV in the PDMS devices with the employed suspending media. This zeta potential value applied to all internal walls of the channel, including the surfaces of the insulating posts. All experiments were carried out with a particle sample solution containing 5.1 µm of red (2.84 × 10^8^ particles/mL) and 2.0 green polystyrene microparticles (4.11 × 10^7^ particles/mL). Particle properties are listed in Table 1. The mobility characterization of the microparticles was performed by employing particle image velocimetry (PIV), a previously established procedure for assessing linear and nonlinear EP particle mobilities [7,23].

### 3.3. Equipment and Software

Particle behavior during EK injection and separation was observed with a ZEISS Axiovert 40 CFL inverted microscope (Carl Zeiss Microscopy, Thornwood, NY, USA). Voltages were applied by employing a high voltage supply (Model HVS6000D, LabSmith, Livermore, CA, USA). The voltage sequencer was controlled with the Sequence software provided by the manufacturer. COMSOL Multiphysics^®^ 4.4 was also used to simulate the electric field distribution across the channel and particle velocities. A description of the mathematical model used in COMSOL and Table S1, which lists the numerical values used with the COMSOL model, are included in the Supplementary Materials.

### 3.4. Experimental Procedures

A particle sample with 10 µL of each particle solution was injected into reservoir A of the channel for a total sample volume of 20 µL (Figure 1a). Then, pressure-driven flow was minimized by balancing the fluid levels in the reservoirs. Voltages were then applied to the four reservoirs, as shown in the sequences in Table 2 following the three-step process of loading, gating, and injection. The run time refers to how long the voltages for a given step were applied before switching to the next voltage step. The run time for the injection step was determined by how long it took the sample plug to fully elute from the post array.

## 4. Results and Discussion

### 4.1. Modeling Predictions of the Effects of Electrophoresis of the Second Kind on Electrokinetic Injections

To analyze the effects of EP^(3)^ on the quality of EK injections, we first modeled overall particle velocities (Equation (8)) by employing COMSOL Multiphysics. To increase the accuracy of our predictions, DEP effects were considered, even though they may have been minor [36]. The details of the COMSOL model (equations and boundary conditions), as well as the value of the numerical parameters employed in the model, are included in the (Supplementary Materials Table S1 and mathematical model description section). The modeling work included the prediction of particle velocities for both particles by employing the mobility data in Table 1 and the voltages listed for the gating step in Table 2 for the “good” and the “bad” EK injections. The overall electric fields resulting from these applied voltages were calculated by dividing the voltage potentials by the distance between reservoirs (Table S2 in Supplementary Materials). The gating step was selected to illustrate the quality of the injection, as this is the step responsible for directing the particle sample into the separation channel. The injection step simply pushes the sample through the separation channel, but its role is not as critical as the role of the gating step.

**Figure 2:**
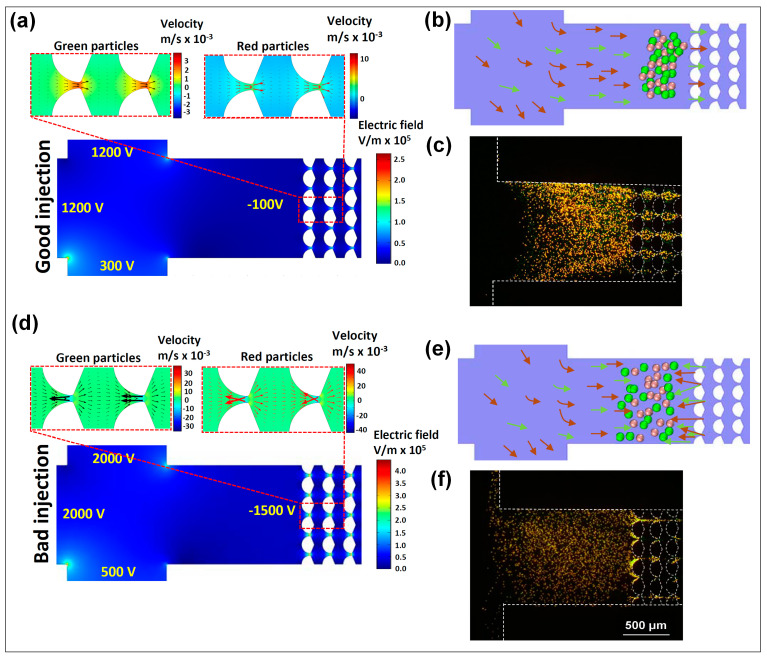
Effects of EP^(3)^ on EK injections. Good EK injection (**a**) simulations in COMSOL Multiphysics depicting electric field distribution at the channel inlet during the gating step, with the two insets above the channel image depicting the direction of overall particle velocity; (**b**) cartoon illustration of the expected overall particle velocity; and (**c**) experimental observation of the particles entering the post array. Bad EK injection (**d**) simulations in COMSOL Multiphysics; (**e**) cartoon illustration of overall particle velocity; and (**f**) experimental observation of the particles entering the post array.

Figure 2 illustrates the effects of EP^(3)^ on both EK injections. The COMSOL simulations (Figure 2a,d) are used to depict the electric field distribution to illustrate that a higher electric field magnitude was reached at the constriction regions between the insulating posts. Nonlinear EK effects became significant in these regions as a result of the higher local electric field magnitude. Additionally, shown in the Figure 2a,d are the overall particle velocities for both particles, depicted in the two insets above the channel image. These insets represent particle velocities as arrows, where the main difference between the “good” and the “bad” EK injections can be seen by observing the direction of the arrows. Under the voltages selected for the good EK injection, the overall particle velocity (Equation (8)) for both particles was towards the post array, that is, the particles were being successfully injected into the channel and could be separated by exploiting the differences in their electromigration. Particle velocity is represented by our COMSOL model as arrows and as the color surface plot. Under the voltages employed for the bad EK injection, it could be observed that particles exhibited negative velocities (velocity toward the inlet, reservoir B) at the entrance of the post array, which caused a significant adverse effect on the quality of the injection by preventing the particle from successfully entering the post array. The velocity insets for both particles clearly depict these negative velocities as predicted by the COMSOL model. Figure 2b,e illustrates cartoons of the behavior expected by both particles in both injections. Furthermore, Figure 2c,f demonstrates that the experimental results matched the predictions made with the COMSOL mathematical model. As predicted, the voltages used for the “good injection” resulted in a successful sample injection where both particles entered the post array. In contrast, the voltages employed for the “bad injection” resulted in particle agglomeration and trapping at the entrance of the post array, thus resulting in a problematic injection.

Excellent agreement was found between modeling and experimental results regarding the direction of particle migration. We attribute the high accuracy of the model to the inclusion of EP^(3)^. Only a handful of reports in the field of microscale EK separations have considered the effects of EP^(3)^ in detail: five recent developments by our group [7,8,23,25,36] and the recent work of Rouhi et al. [37] and Tottori et al. [33]. Until recently, correction factors [38] were commonly added to mathematical models to improve the accuracy of modeling predictions. Considering the effects of EP^(3)^, which are enhanced at the constriction regions between posts due to the higher local electric field magnitude, allows for the first proper design of EK injection schemes by employing mathematical modeling. The modeling and experimental results demonstrated that better gating and injection steps favored lower voltages because they induced lower electric fields that allowed particles to enter the channel while minimizing the effect of EP^(3)^. This is further reinforced in Figure S1, which illustrates velocity profiles of the particles with and without the effects of EP^(3)^ during the gating steps for the good and bad injections. The next section contains an application where the effects of EP^(3)^ on EK injections and particle separations are fully illustrated.

### 4.2. Application: Effects of Electrophoresis of the Second Kind on Particle Separations

**Figure 3:**
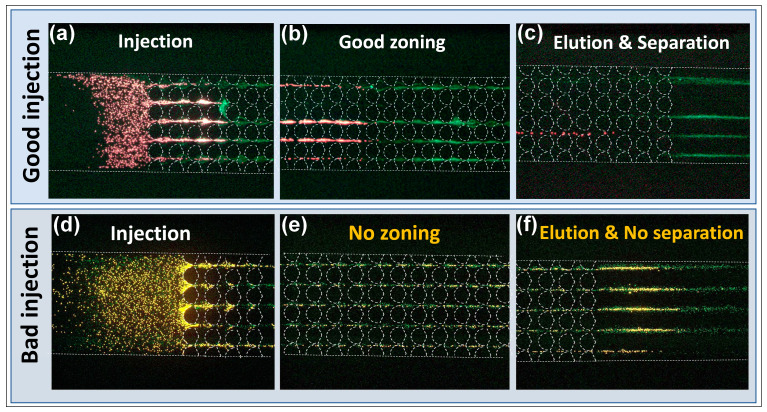
Illustration of the progress of the particle separation for the good and bad injections. Good injection: (**a**) Particles at the start of the post array after injection; (**b**) particles migrating under the streaming regime, creating “zones” at the middle of the post array as they began to separate; (**c**) particles being eluted from the post array, where green particles were eluted first. Bad injection: (**d**) Particles at the start of the post array after injection, where some particle agglomeration and trapping negatively affected the injection process; (**e**) particles at the middle of the post array, where no particle “zones” were observed; (**f**) particles being eluted from the post array, where no separation was observed and both types of particles were eluted together.

To further analyze the effects of EP^(3)^ on EK injections, we experimentally carried out the electrokinetic separations of the two types of microparticles employed in this study (Table 1) by applying the voltages listed in Table 2. These voltages were selected by employing simulations with COMSOL Multiphysics that predicted the electric field distribution, and more importantly, the particle velocity under an applied set of voltages. Mathematical modeling allowed us to identify suitable voltages (particle sample entering the separation channel) prior to experimentation. To fully illustrate good and bad injections, different phases of the two-particle separation were observed at three different locations in the channel: the start of the post array where the particle sample entered the separation channel (Figure 3a,d), within the post array (Figure 3b,e), and at the outlet of the post array (Figure 3c,f). These three phases represent the injection, particle zoning (particle separating into two zones within the post array), and elution of the separated particles. The successful injection depicted in Figure 3a shows particles entering the channel with a smooth flow regime. By comparing Figure 3a,d, it can be seen that for the bad injection, particles initially entering the post array experienced much more resistance from the higher electric field magnitude that resulted in EP^(3)^ forces that pushed some of the particles backwards (as represented in Figure 2e,f), causing them to agglomerate and become trapped between posts. Under the voltages used for the bad injection, there was clear evidence of negative particle velocities for both particles (insets above Figure 2d; particle velocities are represented as arrows illustrating direction). Particle zoning was observed when particles migrating under the streaming regime began to separate into two distinct zones within the post array. For the good injection, as particles migrated under the streaming regime, successful particle zoning where the two particles began to separate was observed. These particle zones and the resulting particle separation were achieved by exploiting small differences in the particle EK mobilities, which impacted particle velocity. The differences in particle velocities, small for each individual constriction between two posts, were amplified at each constriction as particles migrated through the channel, further separating over time due to differences in mobilities. The green particles had a higher overall mobility toward the outlet, as seen in Figure 3b. In contrast, for the bad injection, there was no distinction between two ‘zones’ of particles (Figure 3e), indicating a poor final separation. Finally, in the elution phase, the two types of particles left the post array and could be collected in the outlet. If particle separation was achieved, then a clear stream of one type of particle was observed first, followed by a stream of the second type of particle, as illustrated in Figure 3c. However, if the sample injection was poor, this affected the entire separation process and resulted in a stream of mixed particles being eluted, as shown in Figure 3f for the bad injection.

**Figure 4:**
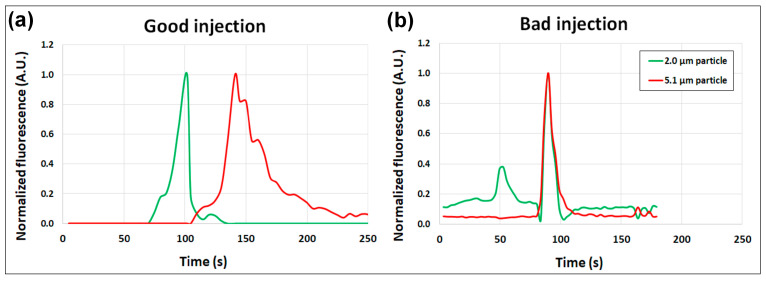
Electropherograms obtained (**a**) a good injection and (**b**) a bad injection. Fluorescence signals were captured at a selected observation window located at the end of the post array, as depicted in Figure 1a.

While observing the particle separating into two “zones” within the post array can be a helpful indicator of how effective the separation would be, the quality of the injection was ultimately quantified by analyzing the resolution of the electropherogram of this separation. As particles were eluted from the post, their fluorescence signal was captured at the interrogation window illustrated in Figure 1. Fluorescence signal analysis was conducted with ImageJ to measure the normalized fluorescence intensity of each particle type as they passed through the interrogation window. The resulting normalized fluorescence signals were plotted as a function of time as the two electropherograms illustrated in Figure 4 for both injections. As can be observed, the good injection resulted in more distinct signal peaks, which indicated a better separation with a separation resolution of Rs = 1.30 and high number of plates—N_1_ = 416 plates and N_2_ = 369 plates for the green and red particles, respectively. The bad injection had a separation resolution of Rs = 0, since there was no separation between the two signal peaks because the peaks completely overlapped with each other, with low number of plates—N_1_ = 199 plates and N_2_ = 135 plates for the green and red particles, respectively. The results in Figure 3 and Figure 4 clearly illustrate how the effects of EP^(3)^ influenced the quality of the injection and thus, the quality of the particle separation. Insulator-based EK (iEK) systems are becoming increasingly important in separation applications due to their robustness and simplicity; therefore, it is essential to be able to properly design EK injection schemes that consider the effects of EP^(3)^ forces.

## 5. Conclusions

Presented here are experimental and simulation data demonstrating the effects of EP^(3)^ on electrokinetic injection and particle separation in insulator-based EK (iEK) systems. As recently demonstrated by several research groups [7,8,23,25,33,36,37], the electrophoresis of the second kind (EP^(3)^) has a dominant effect on particle electromigration at high electric fields in iEK systems. Explicitly, the nonlinear phenomena of EP^(3)^ must be considered when designing a separation process with an iEK system—those that include an EK injection in particular.

As the number of applications of iEK systems is continuously growing, in particular for carrying out a variety of bioparticle separations that include DNA [39], proteins [25], virus [40], and cells [36], it is essential to be able to design effective EK injection schemes. An essential requirement for a successful EK injection is to consider the effects of EP^(3)^. In this study, it was demonstrated with mathematical modeling and experimentation that EP^(3)^ could significantly influence the quality of an EK injection. The effects of EP^(3)^ could produce particle agglomeration and trapping at the inlet of the post array in an iEK device, or they could cause the particles to exhibit a negative (backwards) velocity, thus preventing them from even entering the insulating post array. Furthermore, the modeling predictions were confirmed with experimental results where, for the first time, modeling results had great agreement with experimentation because EP^(3)^ effects were considered. To further illustrate the effect of EP^(3)^, the separation of a two-particle mixture was performed, where particles migrated across the insulating post array under the streaming regime, thus allowing for effective particle separation. The results obtained with the good and bad EK injections were compared using electropherograms. The progress of the particle separations was monitored in three locations along the separation channel, and the final separation results were illustrated as electropherograms. As expected, the results obtained with the good EK injection were superior, in terms of separation resolution (*Rs*) and number of plates (*N*), to those obtained with the bad EK injection (which were affected by greater EP^(3)^ effects during gating). Thus, it was concluded that voltages that create lower electric fields will, in turn, lessen the effects of EP^(3)^ during gating and produce a better separation in terms of resolution and number of plates. Mathematical modeling and simulations are recommended prior to experimentation, as modeling can save significant time and resources, although it is important to consider that there can be some variations when compared to experimental results. Nonlinear forces can also cause unusual particle motion, such as vortices, which could be observed within the system during experimentation. This type of anomalous motion could be caused by the motion of bulk charges. The findings in this study clearly illustrate the importance of considering the nonlinear EK phenomena of EP^(3)^ in designing EK injections and separation processes with iEK systems.

## Figures and Tables

**Table 1:** Detailed information on the microparticles employed in this project [7].

Diameter (µm)	Color	Brand	Surf. Funct.	Concentration (Particles/mL)	$\zeta_{P}$ (mV)	$\mu_{EP}^{\left(1\right)}\times 10^{-9}$ (m^2^V^−1^s^−1^)	$\mu_{EP}^{\left(3\right)}\times 10^{-19}$ (m^4^V^−3^s^−1^)
2.0	Green	Magsphere	Non-funct.	2.84 × 10^8^	−14.6 ± 3.6	−11.3 ± 2.8	−8.5 ± 0.1
5.1	Red	Magsphere	Carboxyl.	4.11 × 10^7^	−7.16 ± 4.0	−5.6 ± 3.1	−9.2 ± 0.4

**Table 2:** Voltages employed for good and bad EK injections.

Step		Applied Voltage (V)			
**Good Injection**	**Run Time (s)**	**Reservoir A**	**Reservoir B**	**Reservoir C**	**Reservoir D**
**Loading**	10 s	300 V	200 V	−200 V	400 V
**Gating**	10 s	1200 V	1200 V	300 V	−100 V
**Injection**	440 s	100 V	1300 V	100 V	−300 V
**Bad Injection**	**Run Time (s)**	**Reservoir A**	**Reservoir B**	**Reservoir C**	**Reservoir D**
**Loading**	10 s	300 V	200 V	−200 V	400 V
**Gating**	10 s	2000 V	2000 V	500 V	−1500 V
**Injection**	340 s	100 V	1500 V	100 V	−500 V

## Data Availability

Data are contained within the article and Supplementary Materials.

## Supplementary Materials

The following are available online at https://www.mdpi.com/article/10.3390/mi12060628/s1. Mathematical Model Description; Table S1: Parameters employed with the COMSOL model; Table S2: Electric field values produced by applied voltages; and Figure S1: Particle velocities during the gating steps for the good and bad injections, with and without the effects of EP^(3)^.
