# Supplementary material for: Fine-Tuning Electrokinetic Injections Considering Nonlinear Electrokinetic Effects in Insulator-Based Devices
Source: Micromachines (Basel). 2021 May 28;12(6):628. doi: 10.3390/mi12060628 (PMC8227112; doi:10.3390/mi12060628)
Supplement: Supplementary file 1 [file micromachines-12-00628-s001.zip › micromachines-1180622-supplementary.pdf]

Supplementary Material

Fine-tuning Electrokinetic Injections Considering Nonlinear Electrokinetic Effects in Insulator-based Devices

Abbi Miller, Nicole Hill, Kel Hakim and Blanca H. Lapizco-Encinas*

Microscale Bioseparations Laboratory and Biomedical Engineering Department, Rochester Institute of Technology, Rochester NY, USA

Contents

Mathematical Model Description 1

Table S1. Parameters employed with the COMSOL model 2

Table S2. Electric field values produced by applied voltages.. 2

Figure S1. Particle velocities during the gating steps for the good and bad injections, with and without the effects of $EP^{(3)}$.. 3

Mathematical Model Description

A microchannel design with an array of asymmetric insulating posts as depicted in Figure 1a. The asymmetric posts are made from the same substrate material employed to fabricate the channel, polydimethylsiloxane (PDMS), and are nonconductive when compare to the liquid suspension medium in the channel. These posts are assumed as insulators that distort the electric field distribution across the channel, creating zones of higher field intensity at the constriction regions between the posts. COMSOL *Multiphysics* 4.4 (COMSOL, Inc., Newton, MA) with the AC/DC module was used to estimate the distribution of the electric field and particle velocities in both channel designs. The COMSOL model built for this study used a mesh of 2,545,800 elements, considering a two-dimensional model, as it has shown that the electric field distributions does not vary

significantly across the channel depth [1].

A brief description of the model is included below. Laplace equation was employed to describe the distribution of the electric potential in the microchannel:

$$\nabla^2 \phi = 0 \qquad \text{(S1)}$$

where φ is the electric potential. The following boundary conditions were considered:

$\vec{n} \cdot \vec{J} = 0$ at the channel boundaries and post boundaries (S2)

$\phi = V_{applied}$ at each one of the four reservoirs of the channel (S3)

where $\vec{n}$ is the normal vector to the surface, $\vec{J}$ is the electrical current density and V_{applied} represent the distinct potentials applied at each one of the microchannel four reservoirs, which are listed in Table 2.

The individual steps of the injection process, loading, gating, and injection, were simulated under a steady-state assumption as the simulations of interest were the behavior of the particles while the particles are within a single step.

Table S1. Parameters employed with the COMSOL model

Table S1. Parameters employed with the COMSOL model.

Parameter	Value
Medium relative permittivity	78.4
Permittivity of vacuum (ε_0)	$8.854 \text{ x}10^{-12}$ [F/m]
Medium permittivity (ε_m)	$78.4 \cdot \varepsilon_0$
Medium viscosity	8.91×10^{-4} [$kg \cdot m^{-1} \cdot s^{-1}$]

Table S2. Electric field values produced by applied voltages

Table S2. Distances between reservoirs and the corresponding electric fields produced by the applied voltages for "good" and "bad" injections

**Distance between reservoirs**	**5.154 cm**	**2.2 cm**	**2.2 cm**	**2.2 cm**	**5.154 cm**	**5.154 cm**
	**Electric field (V/cm)**					
**Good injection**	**A to D**	**A to C**	**A to B**	**B to C**	**B to D**	**C to D**
**Loading**	19	227	45	182	39	116
**Gating**	252	409	0	409	252	78
**Injection**	78	0	545	545	310	78
**Bad injection**						
**Loading**	19	227	45	182	39	116
**Gating**	679	682	0	682	679	388
**Injection**	116	0	636	636	388	116

Figure S1. Particle velocities during the gating steps for the good and bad injections, with and without the effects of EP$^{(3)}$

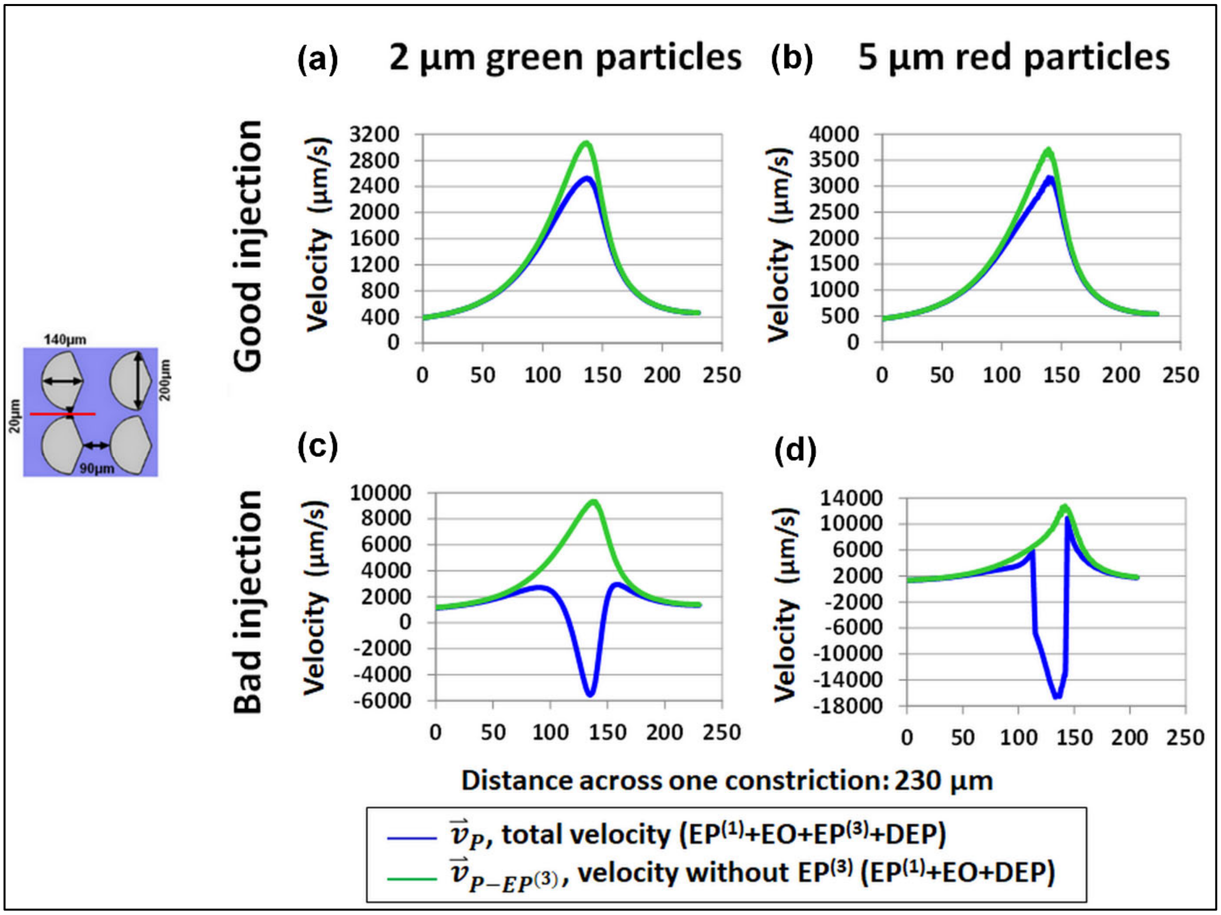

Figure S1. Representations of the total particle velocities simulated in COMSOL **with** and **without** the effects of EP$^{(3)}$ during the gating step for the good and bad injections. Velocity data was obtained along a cutline between two insulating posts, this cutline is depicted in the image included to the left side of the figure. **(a)** Particle velocity for the 2 µm green particles using the voltages for the gating step for the good injection. Note that while the total particle velocity including EP$^{(3)}$ effects has a lower maximum, the overall curves look very similar. **(b)** Particle velocity for the 5 µm red particles using the voltages for the gating step for the good injection. Similar to the 2 µm green particles, the total particle velocity including EP$^{(3)}$ has a lower maximum but the overall curves look very similar. **(c)** Particle velocity for the 2 µm green particles using the voltages for the gating step for the bad injection. In this case, the inclusion of EP$^{(3)}$ shows a dramatic shift in particle behavior. The negative velocities indicate that particles would definitely trap and would not enter the post array as desired. **(b)** Particle velocity for the 5 µm red particles using voltages for the gating step for the bad injection. Similar to the 2 µm green particles in the bad injection, the inclusion of EP$^{(3)}$ shows negative velocities that would lead to particle trapping, which prevents the particle form entering into the post array.

References

[1] Baylon-Cardiel, J. L., Lapizco-Encinas, B. H., Reyes-Betanzo, C., Chávez-Santoscoy, A. V., Martínez-Chapa, S. O., *Lab Chip* 2009, *9*, 2896–2901.
